# Reduced serum VGF levels are linked with suicide risk in Chinese Han patients with major depressive disorder

**DOI:** 10.1186/s12888-020-02634-9

**Published:** 2020-05-12

**Authors:** Xingxing Li, Huifei Ge, Dongsheng Zhou, Xiangping Wu, Gangqiao Qi, Zan Chen, Chang Yu, Yuanyuan Zhang, Haihang Yu, Chuang Wang

**Affiliations:** 1 Ningbo Kangning Hospital, Ningbo, 315201 Zhejiang China; 2 Taizhou 2nd People’s Hospital, Taizhou, 317200 Zhejiang China; 3 Department of Physiology and Pharmacology, School of Medicine, Ningbo University, 818 Fenghua Road, Ningbo, Zhejiang 315211 China

**Keywords:** Major depressive disorder (MDD), VGF, Suicide risk

## Abstract

**Background:**

VGF (nonacronymic) is a neuropeptide that plays an important role in the pathogenesis of major depressive disorder (MDD). However, no studies have yet investigated VGF levels in patients with MDD who are at risk of suicide. The purpose of the present study was to determine whether serum VGF levels are related to suicide risk in patients with MMD.

**Methods:**

A total of 107 patients with MDD and 40 normal control participated in the present study. The risk of suicide was assessed using the Nurses Global Assessment of Suicide Risk (NGASR). On this basis, 60 patients were assigned to a high-risk group (NGASR≥9) and 47 were assigned to a low-risk group (NGASR< 9). The severity of depression was measured using the 17-item Hamilton Depression Rating Scale (HDRS). Levels of serum VGF were determined using a double antibody sandwich enzyme-linked immunosorbent assay.

**Results:**

Serum VGF levels in the high-risk group (883.34 ± 139.67 pg/mL) were significantly lower than in the low-risk group (1020.56 ± 131.76 pg/mL) and in the control group (1107.00 ± 155.38 pg/mL) (F = 31.90, *p* < 0.001). In patients with MDD, suicide risk was significantly negatively correlated with VGF levels (*r* = − 0.55, *p* = 0.001).

**Conclusions:**

Reduced serum VGF levels are related to risk of suicide in patients with MDD, so VGF may be a biomarker of suicide risk in MDD.

## Background

Nationwide, most risk factors continues to have a significant and widespread adverse impact on morbidity and mortality. Suicide risk is elevated after the MDD [1], opioid use disorder [2, 3] or stressful life events [4]. Especially, major depressive disorder (MDD) is a common mental health disorder with high disability and mortality rates [5]. Growing studies have shown that the lifelong incidence of MDD is 17.1, and 15% of patients with MDD die as a result of suicide [6]. Notably, suicidal ideation is considered an important risk factor for suicide; the literature suggests that about a third of depressive patients with suicidal ideation convert to suicidal behavior [7, 8]. However, the mechanisms underlying the suicidal ideation and suicidal behavior of MDD have not been characterized. Therefore, to identify the potential risk of suicide in MDD, it is important to find the biomarkers are linked with suicide risk in Chinese Han patients with MDD.

Suicide is a complex issue involving a number of psychological, social, cultural and biological factors [9]. For example, the levels of 5-hydroxyindole acetic acid (5-HIAA) and hypervanillic acid in the cerebrospinal fluid (CSF) are lower in patients with a history of history of suicidal ideation or history of suicidal behavior than in healthy controls [10, 11].

In addition, there is growing evidence that the link between low cholesterol [12, 13] and orexin [14] and suicidal behavior in people with depression is strongest among those with a history of suicide attempts. In particular, Brundin et al. found a significant negative correlation between orexin levels in the CSF and suicidal symptoms 1 year after attempted suicide in patients with depression [15]. Further, it has been found that prolactin, thyroid hormone, catecholamine, arginine vasopressin, adrenocorticotropin and cortisol are all proposed to be suicide risk in patients with depression levels are associated with suicide attempts in patients with a history of suicidal behavior [16, 17]. More, recent study provide growing evidence that brain-derived neurotrophic factor (BDNF), the most abundant eurotrophin in the brain and plasma, is related to suicidal behaviorin MDD and that BDNF level may be a biological marker of suicidal depression [18]. These results confirm that neurotrophic factors may play an important role in the neurobiology of suicidal behavior and has brought wide attention for the discovery of novel biomarkers indicating the suicide risk of MDD.

Several lines of evidence confirm that neuropeptide *VGF* (nonacronymic) can be induced by both nerve growth factor and neurotrophic factor and may act against depression by regulating the proliferation and survival of neurons [19]. In animal experiments, microinjection of the VGF-derived peptide TLQP62 into the hippocampus of mice produced an anti-depressant-like effect [20], and acquired helplessness and forced swimming in mice could down-regulate VGF protein expression [21]. Autopsies of patients with MDD have found decreased VGF levels in the hippocampus and prefrontal cortex [22], while antidepressants such as ketamine and imipramine increase VGF expression in the hippocampus and prefrontal lobe [22]. Jiang et al. found that serum VGF levels were decreased in patients with MDD, and that antidepressant therapy could reverse this decrease [23]. This was consistent with another study, in which VGF mRNA levels in leukocytes were decreased in patients with MDD and increased during clinically effective antidepressant therapy [24], suggesting that VGF plays an important role in the pathophysiology of MDD.

However, to our knowledge, no previous studies have investigated the relationship between suicide risk and VGF levels in the peripheral blood of patients with MDD. The aim of the present study was to explore whether serum VGF levels were linked to risk of suicide, and thus to determine whether VGF could be used as a biomarker for predicting the risk of suicide in patients with MDD. The ultimate goal of our work is to understand the neurotrophic hypothesis of MDD that contribute to the risk of suicidal behavior.

## Methods

### Subjects and assessment

Between January 2016 and December 2018, 107 patients with MDD were recruited (48 men and 59 women aged 18–65 years). All were patients who had been admitted to a mental health facility in Ningbo, China, and they all met the diagnostic criteria of the Diagnostic and Statistical Manual of Mental Disorders (DSM-V). Trained psychiatrists assessed depression severity using the 17-item Hamilton Depression Rating Scale (HDRS). All patients discontinue antidepressant use for at least 2 weeks. To qualify for the study, the patients had to be able to communicate, understand the purpose of the study, and agree to participate.

The risk of suicide in patients with depression was assessed using the nurse’s global assessment of suicide risk (NGASR) [25]. Patients with an NGASR score ≥ 9 (*n* = 60; 25 men, 35 women) were sorted into the high-suicide-risk group, while those with an NGASR score < 9 (*n* = 47; 23 men, 24 women) were sorted into the low-suicide-risk group. In the high-risk group, all patients had a history of suicidal ideation and suicide attempts. In the low-risk group, none of the patients had ever attempted suicide. The control group consisted of 40 volunteers (18 men, 22 women). They were excluded from the study if they had an HDRS score > 7 points, a state–trait anxiety scale score ≥ 40, any self-reported personal or family history of mental illness, or any personal history of psychotropic drug treatment. The control group matched the patient groups in terms of age, gender, and other parameters.

The exclusion criteria were as follows: severe somatic disease, ethanol or drug dependency, pregnancy or lactation, drug allergies. All subjects participated in the study voluntarily and signed the written informed consent forms. The present study was approved by the Ethics Committees of Ningbo Kangning Hospital. All experimental procedures followed the Declaration of Helsinki guidelines for human medical research.

### Procedures

All subjects completed the HDRS and NGASR, and all were diagnosed and rated by two trained psychiatrists. Venous blood samples (10 mL) were collected in the morning following an overnight fast and centrifuged for 5 min at 3500 rpm; the serum was extracted and stored at − 80 °C to await biochemical analysis.

The serum sample was taken out from the − 80 °C freezer, dissolved and subjected to enzyme-linked immunosorbent assay (ELISA). Serum levels of VGF were tested using VGF [SEB166Hu] ELISA kits from USCN Life Science (Wuhan, Hubei, China). We performed all experiments in duplicate, and every empty added 100 μl sample. Absorbance readings were measured at 450 nm using a microplate reader (EnSpire; Perkin Elmer Inc., Waltham, MA), with a reference wavelength of 690 nm; the readings were then converted into concentrations by comparison with standard curve values.

### Statistical analysis

Statistical analysis of the data was carried out using SPSS 22.0 software. All data are presented as mean ± SD; *p*-values are two-tailed, with statistical significance being set at *p* < 0.05. Demographic and clinical information was analyzed using one-way analysis of variance (ANOVA) for continuous variables and the chi-square test for categorical variables. By controlling factors such as age and education, analysis of covariance was used to determine the differences between the mean values of the groups. VGF levels were compared post hoc between groups. VGF values that deviated from the average value by more than three standard deviations were defined as outliers. In patients with MDD, we further analyzed the correlation between serum VGF and the following clinical variables using Pearson’s correlation: HDRS, NGASR, and demographic and clinical variables.

## Results

**Fig. 1:**
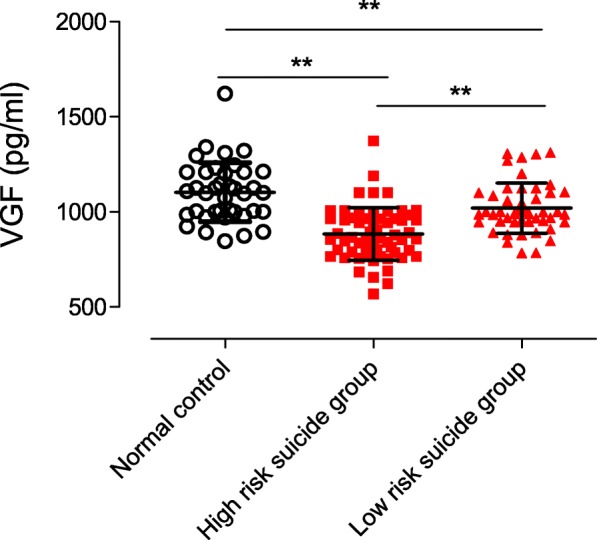
Peripheral concentration of VGF. Serum concentrations of VGF in high risk suicide, low risk suicide and control group were 883.34 ± 139.67 pg/ml, 1020.56 ± 131.76 pg/ml and 1107.00 ± 155.38 pg/ml, respectively (P < 0.01)

**Fig. 2:**
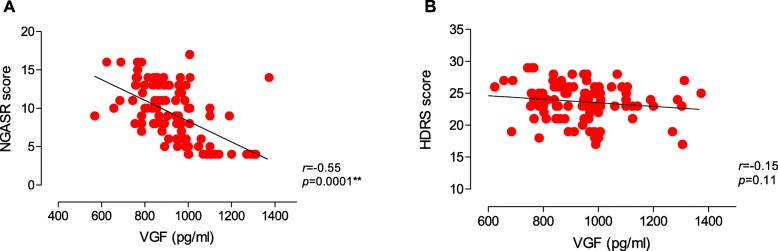
The peripheral VGF concentration correlates to suicide risk and severity of depression. **a** The peripheral VGF concentration have a significant negative correlation to the suicide risk in depression group. **b** The peripheral VGF concentration does not correlate to the severity of depression

The demographic and neuropsychological characteristics of all 107 patients with MDD and 40 healthy controls are shown in Table 1. Among the patients with MDD, 56.07% were placed in the high-risk group. There were no significant differences in gender, age, duration of illness or education among the groups, respectively (F = 0.563, *p* = 0.755; X^2^ = 2.208, *p* = 0.114; F = 0.411, *p* = 0.523; F = 0.637, *p* = 0.531). Furthermore, HDRS score did not differ between the high-risk and low-risk groups (F = 2.748, *p* = 0.100; Table 1). The levels of VGF in the high-risk group (883.34 ± 139.67 pg/mL) were significantly lower than in the low-risk group (1020.56 ± 131.76 pg/mL) and in the normal control group (1107.00 ± 155.38 pg/mL; F = 31.90, *p* < 0.001). The post-hoc test showed significant differences between the high-risk group and both other groups (*p* < 0.001 in both cases), and levels in the low-risk group were significantly lower than those in the normal control group (*p* = 0.05; Table 1, Fig. 1). Correlation analysis showed that the level of serum VGF was negatively correlated with risk of suicide in patients with MDD (*r* = − 0.55, *p* = 0.001), but not with the severity of depression (HDRS score) (*r* = − 0.15, *p* = 0.11; Table 2, Fig. 2).

**Table 1:** Characteristics of MDD patients and healthy control

	MDD (*N* = 107)				
	Normal Control (*N* = 40)	High risk suicide group (*N* = 60)	Low risk suicide group (*N* = 47)	F/X^2^	P
Age (years)	42.02 ± 7.26 ^b^	43.17 ± 11.15 ^a^	39.15 ± 11.11 ^c^	2.208	0.114
Gender (m/f)	18/22 ^b^	25/35 ^a^	23/24 ^c^	0.563	0.755
Education (years)	10.63 ± 6.06 ^b^	11.16 ± 6.50 ^a^	9.43 ± 5.69 ^c^	0.637	0.531
Duration of illness (months)	_	37.70 ± 23.36	34.55 ± 25.11 ^c^	0.411	0.523
HDRS	3.85 ± 1.51 ^b**^	24.05 ± 2.52 ^a**^	23.17 ± 2.96 ^c^	949.17	0.0001**
NGASR		11.88 ± 2.30	5.51 ± 1.53 ^c**^	267.63	0.0001**

*MDD* Major depressive disorders, *HDRS* 17-item Hamilton Depression Rating Scale, *NGASR* Nurses Global Assessment of Suicide Risk

^a^Controls vs. “High risk suicide group” by ANOVA analysis except for gender (chi-square test)

^b^Controls vs. “Low risk suicide group” by ANOVA analysis

^c^“High risk suicide group” vs. “Low risk suicide group” by ANOVA analysis

*P < 0.05, **P < 0.01

**Table 2:** Correlations among Serum VGF levels, HDRS and NGASR scores on in risk suicide depression patients

Parameters	VGF		HAMD	
	R Value	*P* Value	R Value	*P* Value
NGASR	−0.55	0.001**	0.17	0.088
HAMD	−0.15	0.11	–	–
VFG	–	–	− 0.15	0.11

*MDD* Major depressive disorders, *HDRS* 17-item Hamilton Depression Rating Scale, *NGASR* Nurses Global Assessment of Suicide Risk

*P < 0.05, **P < 0.01

## Discussion

To the best of our knowledge, this was the first study to address the relationship between suicide risk and serum VGF levels in patients with MDD. Our present findings indicate that the risk of suicide in patients with MDD was closely related to the level of serum VGF.

Herein, our current study further showed that serum VGF levels were significantly lower in patients with acute MDD who were not taking drugs than in the control group, indicating that peripheral VGF level is decreased in MDD. Our current data consistent with the previous experiment showed that levels of VGF in the peripheral blood were lower in patients with MDD than in healthy controls and after treatment with the antidepressants escitalopram and duloxetine, the levels of serum VGF were recovered [23], indicating that the serum VGF may reflect the changes of MDD symptoms. It is not clear whether VGF passes through the blood-brain barrier, but it has been found in certain brain regions, as well as in CSF [26]. These results show that VGF plays an important role in the neurobiology and pathophysiology of MDD.

To the best of our knowledge, none study have shown the relationship between the risk of suicide and VGF levels. In order to confirm whether VGF changes to reflect the risk of suicide in MDD. Our study further found that patients with high suicide risk had lower serum VGF than those at low risk, and that the risk of suicide was negatively correlated with the level of VGF. Consistent with our present study, previous studies demonstrated that the lower levels of neuropeptide Y in the prefrontal cortex and caudate nucleus of suicide victims [27], and Kang et al. found that hypermethylation of brain-derived neurotrophic factor (BDNF) plays a role in the epigenetic susceptibility of patients with acute coronary syndrome who have a history of suicidal ideation [28]. Taken together, these studies suggest that the VGF as one critical neuropeptides is also closely related to suicide risk.

What mechanism leads to the serum VGF significantly reduced in MDD? VGF, being a neuropeptide, may therefore play a key role in the human stress response. Stress events such as attempted suicide may alter the responsiveness of the hypothalamic-pituitary-adrenal system, and stress-induced corticosteroids reduce the expression of VGF levels in the serum and brain [29]. Stress events can also enhance the expression of tyrosine hydroxylase, which regulates catecholamine synthesis, while glycopeptide increases with the final increase of norepinephrine transmission [30, 31]. This may determine the direct and indirect inhibition of the release or expression of VGF. In addition, another explanation for the low levels of VGF in patients at risk of suicide is that decreased serotonin function in such patients may down-regulate the expression of VGF. Patients with suicidal depression have lower levels of 5-HIAA, which is the main decomposition product of serotonin in the CSF [32], indicating that low levels of CSF 5-HIAA predict future suicides and attempted suicides. Other indicators of decreased serotonin function in suicidal depression include prolactin insensitivity to fluoroamphetamine and abnormal platelet serotonin function [33]. These two signaling molecules are related to each other, while VGF and BDNF regulate each other [34]. Therefore, a damaged serotonin signal may reduce VGF expression in patients with suicidal depression.

However, our present study may have some limitations. Firstly, the sample size was very small. Secondly, it is not clear whether VGF can cross the blood-brain barrier or whether VGF levels in the peripheral blood represent levels in the brain. Thirdly, to highlight the specificity of our findings in patients with suicidal depression, we only tested VGF levels; we did not detect other neurotrophic factors that have been associated with suicide risk. In the future studies, we will evaluate serum VGF levels in a large number of matched control subjects; we will also conduct longitudinal studies to explain these preliminary findings.

## Conclusion

The results of our current study showed that VGF levels in patients with MDD were down-regulated, and that the risk of suicide is associated with decreases of VGF levels. This report, documenting VGF may be a biomarker of suicide risk in MDD. In the future, the use of serum VGF levels for suicide may be a relevant tool to assist in the diagnosis of MDD. This will facilitate diagnosis and early treatment to reduce suicide mortality rates of MDD.

## Data Availability

The datasets used and/or analyzed during the current study are available from the corresponding author on reasonable request.

## Abbreviations

MDD: Major depressive disorder
CSF: Cerebrospinal fluid;
BDNF: Brain-derived neurotrophic factor
NGASR: Nurse’s global assessment of suicide risk
